# Medizinstudierende als Helfende in der Pandemie: Innovatives Konzept zu Rekrutierung, Schulung und Einsatzplanung von Medizinstudierenden als medizinisches Personal während der COVID-19-Pandemie

**DOI:** 10.1007/s00101-021-01009-3

**Published:** 2021-07-20

**Authors:** Lina Vogt, Michelle Schmidt, Martin Klasen, Johannes Bickenbach, Gernot Marx, Saša Sopka

**Affiliations:** 1 Klinik für Anästhesiologie, Uniklinik RWTH Aachen, Medizinische Fakultät, RWTH Aachen, Aachen, Deutschland; 2 AIXTRA – Kompetenzzentrum für Training und Patientensicherheit, Medizinische Fakultät, RWTH Aachen, Forckenbeckstraße 71, 52074 Aachen, Deutschland; 3 Klinik für Operative Intensivmedizin und Intermediate Care, Uniklinik RWTH Aachen, Medizinische Fakultät, RWTH Aachen, Aachen, Deutschland

**Keywords:** Patientensicherheit, Intensivmedizin, Pandemiemanagement, Personalakquise, Personalentwicklung, Patient safety, Intensive care medicine, Pandemic management, Personnel acquisition, Personnel development

## Abstract

**Hintergrund:**

Die COVID-19-Pandemie hat das deutsche Gesundheitssystem vor enorme Herausforderungen gestellt und den Bedarf an Strategien zu Rekrutierung, Schulung und Einsatzplanung von medizinischem Personal verdeutlicht. Bislang existierte kein ganzheitliches Konzept, welches Medizinstudierende als Unterstützung der Fachkräfte auf Intensivstationen (ICU) einsetzt, um Personalengpässe in der medizinischen Versorgung zu vermeiden.

**Methode:**

In einem groß angelegten Pilotprojekt wurden 265 Medizinstudierende für den Einsatz auf ICU trainiert. Begleitet wurde das innovative Schulungsmodul mit einem Prä-post-Fragebogen zur Selbsteinschätzung über die erlernten Fertigkeiten. 22 Wochen nach dem Schulungsmodul und noch während des Pandemieeinsatzes wurden mit einem weiteren Fragebogen Erfahrungen im Einsatz und Effizienz des Trainingsmoduls in Bezug auf die Vorbereitung für den ICU-Einsatz evaluiert.

**Ergebnisse:**

Die Analyse ergab signifikante Mittelwertdifferenzen für alle COVID-19-spezifischen Variablen (*Sicherheitsdimension*) zugunsten des Schulungsmoduls (*n* = 168). Die Einsatzevaluation zeigte für *n* = 69 von insgesamt 89 eingesetzten Studierenden eine uneinheitliche Bewertung des Schulungskonzeptes als Vorbereitung auf den Arbeitseinsatz (53 % Zustimmung/47 % Ablehnung).

**Schlussfolgerung:**

Die Ergebnisse zeigen eine gute Realisierbarkeit des innovativen Trainingskonzeptes für Medizinstudierende in Bezug auf einen Pandemieeinsatz als Helfende auf Intensivstationen. Das Konzept ist geeignet, um zusätzliche Hilfskräfte während einer Pandemie auf den Intensivstationen zur Verfügung zu stellen. Die uneinheitliche Bewertung deutet jedoch auf Anpassungsbedarf des Konzeptes hin.

**Zusatzmaterial online:**

Zusätzliche Informationen sind in der Online-Version dieses Artikels (10.1007/s00101-021-01009-3) enthalten.

## Abstract (en)

**Background:**

The COVID-19 pandemic posed enormous challenges to the German healthcare system and highlighted the need for strategies to recruit, train, and deploy medical personnel. Until now, no holistic concept existed to use medical students as support for professionals in intensive care units (ICU) to avoid staff shortages in medical care.

**Method:**

In a large-scale pilot project 265 medical students were trained for an ICU assignment. The innovative training module was accompanied by a pre-post questionnaire for self-assessment of the skills learned. 22 weeks after the training module and still during the pandemic deployment, another questionnaire was used to evaluate experiences in deployment and the efficiency of the training module with respect to preparation for ICU deployment.

**Results:**

The analysis revealed significant mean differences for all COVID-19-specific variables (safety dimension) in favor of the training module (*n* = 168). The deployment evaluation showed that the training concept was inconsistently assessed as preparation for the work deployment for 69 of the 89 deployed students in total (53% agreement/47% disagreement).

**Conclusion:**

The results show a good feasibility of an innovative training concept for medical students with respect to a pandemic deployment as assistants in intensive care units. The concept is suitable for providing additional helpers in intensive care units during a pandemic; however, the inconsistent evaluation indicates that the concept can be expanded and needs to be adapted.

Mit Veröffentlichung der Leitlinie „Empfehlungen zu Schulungen von Mitarbeitenden im Gesundheitswesen bei Einsatz während der COVID-19-Pandemie“ der Deutschen Gesellschaft für Anästhesiologie und Intensivmedizin (DGAI) wächst der Bedarf nach einem ganzheitlichen Konzept, das die Empfehlungen in die Praxis überführt und den Anforderungen der Pandemie gerecht wird. Anhand der vorliegenden Studie wurde ein innovatives Konzept zu Rekrutierung, Schulung und Einsatzplanung von Medizinstudierenden als freiwillige Helfende auf Intensivstationen während der COVID-19-Pandemie entwickelt und evaluiert.

## Einleitung

Seit Anfang 2020 breitet sich das neuartige Coronavirus SARS-CoV‑2 pandemisch aus [1]. Im deutschsprachigen Raum gab es in den letzten Jahrzehnten hinsichtlich Fallzahlen, schwerer Krankheitsverläufe und Ausbreitungsgeschwindigkeit kein vergleichbares Ereignis. Dementsprechend gravierend sind die Auswirkungen auf das Gesundheitssystem, das binnen kürzester Zeit auf die Pandemie reagieren muss. Strukturen, Prozesse und Abläufe des Gesundheitswesens wurden in der Erwartung steigender COVID-19-Patient*innenzahlen angepasst, um die medizinischen Systeme nicht zu überlasten [2–5]. Dabei besteht nicht nur die Gefahr, COVID-19-Patient*innen aufgrund fehlender Ressourcen insuffizient zu versorgen, sondern auch alle diejenigen, die sonstige medizinische Hilfe benötigen.

Um auf eine stark steigende Anzahl der COVID-19-Patient*innen in der Bundesrepublik Deutschland vorbereitet zu sein, medizinischen Versorgungsengpässen vorzubeugen und eine Erweiterung der intensivmedizinischen Kapazitäten zu ermöglichen, wurden erforderliche infrastrukturelle Maßnahmen (z. B. Personalrekrutierung [6], zusätzliches medizinisches Equipment) von Fachgesellschaften in Leitlinien gefordert [7–9]. Die Entwicklung eines national anwendbaren Konzepts zu Rekrutierung und Qualifizierungskonzept zusätzlichen medizinischen Personals nimmt dabei eine Schlüsselrolle ein. Bereits vor der Pandemie bestand in Deutschland ein Defizit von ca. 9000 fehlenden Pflegekräften [10, 11] sowie von ca. 30.000 unbesetzten Stellen in der Alten‑, Gesundheits- und Krankenpflege [10]. Angesichts einer derart angespannten Personalsituation war und ist der Bedarf nach einem kurzfristig umsetzbaren Rekrutierungs- und Qualifikationskonzept immens.

Neben der Möglichkeit, ehemalige medizinische Fachkräfte und medizinisch gering qualifiziertes Hilfspersonal zu aktivieren, existiert an universitären Standorten die Ressource der Medizinstudierenden, die über theoretisches Wissen und gewisse praktische Kompetenzen verfügen. Bundesweit existieren ca. 30.000 Studierende mit abgeschlossenem 1. Abschnitt der ärztlichen Prüfung (M1), die ein bis dato ungenutztes Potenzial darstellen [12].

Ziel dieses Pilotprojektes war, das innovative Konzept der DGAI [7] zur Schulung von medizinischen Helfenden beim Einsatz während der COVID-19-Pandemie zu evaluieren und Empfehlungen für den Transfer auf andere Standorte abzuleiten.

Primäre Fragestellung war, den Einfluss eines innovativen Schulungsmoduls auf das Sicherheitsgefühl der Studierenden sowie Einschätzungen und Erfahrungen nach dem Einsatz auf der Intensivstation zu erheben. Hierbei waren insbesondere das Sicherheitsgefühl an einem intensivmedizinischen Arbeitsplatz und die Bedienung von Beatmungsgeräten zentrale Aspekte, da diese eine Schlüsselrolle in der Bewältigung der COVID-19-Pandemie einnehmen. Sekundär wurde evaluiert, welchen nachhaltigen Charakter ein solches Konzept für die flexible zielgerichtete Qualifizierung von medizinischem Personal hat.

## Methoden

### Ablauf und Datenerhebung

In einem Pilotprojekt wurden 265 Medizinstudierende für den Einsatz auf Intensive Care Units (ICU) als Assistenz des medizinischen Fachpersonals trainiert. Begleitet wurde das innovative Schulungsmodul mit einem Prä-post-Fragebogen zur Selbsteinschätzung hinsichtlich der erlernten Fertigkeiten. 22 Wochen nach dem Schulungsmodul und noch während des Pandemieeinsatzes wurden Erfahrungen im Einsatz und die effiziente Vorbereitung durch das Trainingsmodul evaluiert.

### Rekrutierung

Die Kontaktaufnahme mit ca. 1200 Studierenden erfolgte vom 25.03.2020 bis 16.04.2020 mittels E‑Mail. Hierbei wurden berufliche Qualifikationen im medizinischen Bereich sowie Verfügbarkeit erfragt.

Als Haupteinschlusskriterium für die Rekrutierung und Einladung zur Schulung galt der fachliche Hintergrund. Dieser wurde sichergestellt, indem Medizinstudierende ab dem 8. Fachsemester sowie Medizinstudierende mit Abschluss in der Gesundheits- und Krankenpflege oder als Notfallsanitäter eingeschlossen wurden. Darüber hinaus war die Verfügbarkeit in den kommenden 6 Monaten ein notwendiges Einschlusskriterium. Insgesamt erfüllten 265 Freiwillige die Einschlusskriterien und nahmen an der Schulung teil.

Die Untersuchung wurde von der unabhängigen Ethik-Kommission der RWTH Aachen genehmigt (EK-Nummer 184-20).

### Schulung

Eine interdisziplinäre Expertengruppe aus Pflegedirektion, ärztlicher Leitung der operativen Intensivmedizin, Patientensicherheit und medizinischer Ausbildung erarbeitete Lernziele und praktische Inhalte des Schulungskonzepts. Die Schulung fand an einem Tag im Umfang von 5 h statt. Die erste Hälfte der Schulung (2,5 h) bestand aus theoretischer Schulung in Form von interaktiven Kurzvorträgen. Die hierbei behandelten Themen waren Besonderheiten von COVID-19, respiratorische Insuffizienz, Beatmungsformen und Respiratoreinstellungen, Checklisten (FAST HUG), Hygienemaßnahmen, Kommunikation und basale Aspekte von Crisis Resource Management (CRM) sowie Dokumentation. Die zweite Hälfte der Schulung (2,5 h) bestand aus praktischen Trainingseinheiten in Kleingruppen an 5 Stationen mit jeweils unterschiedlichem Themenschwerpunkt: ICU-Arbeitsplatz, Respiratoreinstellungen, ICU-Monitoring Vitaldaten, Vorbereitung Bettplatz und Infusion sowie Materialkunde/steriles Arbeiten. Bei den praktischen Einheiten wurden die zu lernenden Fertigkeiten zunächst von den Anleitenden demonstriert und anschließend überprüft. Der Aufbau der 5‑stündigen Schulung folgte den DGAI-Empfehlungen [7]. Intensivmedizinische Basiskompetenzen wurden in theoretischen und praktischen Modulen geschult, von Hygienemaßnahmen über Kommunikation und Checklisten bis hin zu Einstellungen von Beatmungsgeräten, Vital- und Monitorparametern. Hervorzuheben ist, dass die Medizinstudierenden in der Schulung auf primär assistierende Aufgaben vorbereitet wurden. Dies umfasst beispielsweise das Anreichen von Material für Pflegekräfte, die in der Isolationsbox tätig sind. Alle Tätigkeiten fanden stets unter Supervision von examiniertem Pflegepersonal statt. Eine genaue Rückverfolgung, welche spezifischen Tätigkeiten die einzelnen Studierenden in ihrem jeweiligen Einsatz durchführten, ist nicht Bestandteil dieser Studie.

Zum Schutz und zur Sicherheit der Studierenden und Dozierenden erfolgten die Schulungsmaßnahmen unter strengen Hygienemaßnahmen, wie ausreichendem Abstand > 2 m (große Räumlichkeiten), Mund-Nasen-Schutz, Händedesinfektion, Kleingruppen (max. 6 Personen), gesonderte Wegeführung u. v. m.

Nach der Schulungsmaßnahme übermittelten die Studierenden ihre Stammdaten zur Vertragserstellung mittels QR-Codes. Die Personalabteilung kontaktierte die Studierenden zur Unterzeichnung der Pflegehelferverträge; die Pflegedirektion koordinierte den Arbeitseinsatz auf den Intensivstationen.

Bei der Schulung handelte es sich um eine freiwillige, unbezahlte Veranstaltung. Angesichts steigender Infektionszahlen und des damit verbundenen immensen Zeitdrucks, schnell zu handeln, fand die Schulung noch vor der Einstellung der Medizinstudierenden statt. Hierbei gilt es zu beachten, dass der Einstellungsprozess immer einen gewissen Zeitraum in Anspruch nimmt, sodass es unter Pandemiebedingungen nicht zu rechtfertigen war, mit der Schulung bis nach der Einstellung zu warten. Darüber hinaus war der genaue Bedarf an intensivpflegerischer Assistenz zu diesem Zeitpunkt nicht absehbar. Die breit angelegte Schulung ermöglichte somit den Aufbau eines Personen-Pools, auf den je nach Bedarf zugegriffen werden konnte. Ein weiterer Aspekt war pragmatisch-ökonomischer Natur. Aufgrund des beachtlichen organisatorischen und personellen Aufwands der Schulung erschien es sinnvoll, die Schulung blockweise in einem kurzen Zeitfenster durchzuführen, in welchem die Dozierenden und Praxisanleitenden auf den Intensivstationen noch nicht unentbehrlich waren. Administrative Prozesse wurden schnellstmöglich geklärt, sodass der Einstellung nach erfolgter Schulung nichts entgegenstand.

Zum damaligen Zeitpunkt bestand ein äußerst kurzfristiger und drastischer Bedarf nach zusätzlichem Personal auf den Intensivstationen. Da die Studierenden im Rahmen vergleichbarer Einsätze und Schulungen, wie beispielsweise während der Famulatur oder dem Blockpraktikum, über ausreichenden Versicherungsschutz verfügen und die Schulungsmaßnahme die gleichen Kriterien erfüllte, ist hier von ebenfalls ausreichendem versicherungsrechtlichen Schutz für die Studierenden auszugehen.

### Fragebogen

In insgesamt 3 Fragebogen (t0, t1, t2) wurden die Medizinstudierenden zu ihrer subjektiven Einschätzung hinsichtlich der im Folgenden beschriebenen Aspekte befragt. Alle Fragebogen beinhalteten Items zur subjektiven Sicherheit in der Ausübung 9 COVID-19-relevanter klinischer Fertigkeiten, die an die Übungsmodule des Schulungskonzeptes angepasst waren (Dimension *Sicherheit*). Der Präfragebogen (t0, 20 Items) erfragte darüber hinaus persönliche Lehr-Lern-Präferenzen. Der Postevaluationsfragebogen (t1) bestand aus 22 Items.

Die dritte Befragung (t2, 26 Items) fand während des Einsatzes statt und beinhaltete zusätzlich die 4 Dimensionen *Belastung *(6 Items), *Integration ins Team *(6 Items), *Sinnhaftigkeit* (3 Items) und *Schulungskonzept* (ein Item). Alle Fragebogen verwendeten eine 6‑stufige Likert-Skala von 1: „stimme überhaupt nicht zu“ bis 6: „stimme voll und ganz zu“. Eine vollständige Übersicht der Fragebogen findet sich im Zusatzmaterial online des Artikels (s. Box am Anfang).

### Statistische Analysen

Die Evaluation des Schulungsmoduls (t0, t1) wurde mittels messwiederholtem t‑Test analysiert. Die Einsatzevaluation wurde deskriptiv analysiert (Mittelwerte und Standardabweichungen sowie Zustimmungs- und Ablehnungswerte (in %) mittels Dichotomisierung der Items). Mittels t‑Test für unabhängige Stichproben wurde untersucht, ob sich diejenigen Studierenden, die sich durch die Schulung adäquat auf den Einsatz vorbereitet fühlten, hinsichtlich der Sicherheitsdimension von denjenigen unterschieden, die sich unzureichend vorbereitet fühlten. Signifikanzschwelle für alle Analysen war *p* < 0,05 (zweiseitig).

Um den Weiterentwicklungsbedarf der Schulung zu untersuchen, wurden die Studierenden basierend auf ihrer Einschätzung des Schulungskonzepts in 2 Gruppen unterteilt („Schulung hat mich gut vorbereitet“ vs. „Schulung hat mich nicht gut vorbereitet“). Diese beiden Gruppen wurden mittels t‑Tests für unabhängige Stichproben hinsichtlich der COVID-19-spezifischen Items (Dimension *Sicherheit*) verglichen, um mögliche Gruppenunterschiede hinsichtlich der subjektiven Sicherheit bei den verschiedenen Tätigkeiten aufzuzeigen.

## Ergebnisse

### Stichprobe

**Figure 1:**
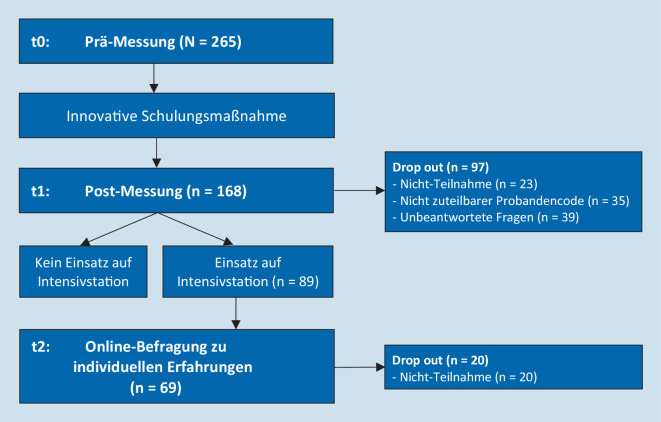

Vor der Schulung nahmen *n* = 265 Studierende an der Befragung teil. An der zweiten Befragung nahmen 23 Personen weniger teil. 35 Datensätze wurden aufgrund von nichtzuteilbaren Probandencodes und 39 Datensätze aufgrund lückenhafter Daten ausgeschlossen (resultierend in *n* = 168, Abb. 1).

Insgesamt wurden *n* = 89 Studierende auf den Intensivstationen eingesetzt. An der dritten Befragung nahmen *n* = 69 Studierende teil (77,53 %).

### Schulungsevaluation

#### Deskriptive Statistiken

Das Sicherheitsgefühl bei der Bedienung eines Respirators vor der Schulung wies den niedrigsten Mittelwert auf (M = 1,73/Ablehnung). Nach der Schulung wies die Bewertung der Fertigkeit, benötigte Materialien für eine Intubation selbst zusammenzustellen (M = 3,27), den geringsten Mittelwert auf. Sowohl vor (M = 4,57) als auch nach der Schulung (M = 5,27) fühlten sich die Medizinstudierenden am sichersten, ein Monitoring bei einem Intensivpatienten anzulegen.

#### Prä-post-Vergleich

Der Prä-post-Vergleich zeigte signifikante Mittelwertdifferenzen für alle COVID-19-spezifischen Items auf (alle *p* < 0,001).

Zusammenfassend hatte die Schulung einen signifikant positiven Einfluss auf das Sicherheitsgefühl bei der Ausführung medizinischer Aufgaben. Nach der Schulung schätzten die Medizinstudierenden ihre Bereitschaft und Sicherheit besser ein als vor der Schulung (Tab. 1).

**Table 1:** 

		t0		t1			
Item	Subjektive Selbsteinschätzungen zu COVID-19 spezifischen Items	M	SD	M	SD	t	df
1	Ich fühle mich bereit, Monitoring (EKG, Sättigung und Blutdruck) bei einem Intensivpatienten anzulegen	4,57	1,45	5,27	0,97	7,58***	167
2	Ich fühle mich bereit, die angezeigten Monitoring-Kurven zu interpretieren	3,82	1,37	4,96	0,84	13,05***	167
3	Ich fühle mich bereit, auf Monitoring-Alarme adäquat zu reagieren	3,01	1,29	4,64	1,06	16,19***	167
4	Ich fühle mich bei der Arbeit an einem intensivmedizinischen Arbeitsplatz sicher	2,42	1,26	3,79	1,15	15,78***	167
5	Ich fühle mich bei der Bedienung eines Respirators sicher	1,73	1,04	3,71	1,14	21,30***	167
6	Ich fühle mich bereit, adäquat auf Alarme des Respirators zu reagieren	1,83	1,11	3,63	1,18	18,04***	167
7	Ich fühle mich bei der Vorbereitung einer Infusion sicher	3,94	1,66	4,67	1,22	7,67***	167
8	Ich fühle mich bereit, die Materialien zur Vorbereitung einer ZVK-Anlage selbst zu übernehmen	2,54	1,53	4,30	1,17	16,12***	167
9	Ich fühle mich bereit, die benötigten Materialien für eine Intubation selbst zusammenzustellen	2,90	1,59	3,27	1,53	4,59***	167

*n* = 168; ***p < 0,001; *t0* vor der Schulung, *t1* nach der Schulung

*M* Mittelwert, *SD* Standardabweichung, *t* Prüfgrößenwert des t‑Tests für abhängige Stichproben, *df* Freiheitsgrade

### Einsatzevaluation

#### Deskriptive Statistiken

Die deskriptiven Statistiken der Einsatzevaluation sowie Ablehnung und Zustimmung zu den jeweiligen Items (in %) zeigt Tab. 2.

**Table 2:** 

		t2			
Item	Subjektive Selbsteinschätzungen	M	SD	Ablehnung (%)	Zustimmung (%)
*Sicherheit*					
1	Ich fühlte mich sicher, Monitoring (EKG, Sättigung und Blutdruck) bei einem Intensivpatienten anzulegen	5,37	1,13	7,4	92,6
2	Ich fühlte mich sicher, die angezeigten Monitoring-Kurven zu interpretieren	4,85	1,18	11,9	88,1
3	Ich fühlte mich sicher, auf Monitoring-Alarme adäquat zu reagieren	4,44	1,42	23,5	76,5
4	Ich fühlte mich bei der Arbeit an einem intensivmedizinischen Arbeitsplatz sicher	4,21	1,14	22,1	77,9
5	Ich fühlte mich bei der Bedienung eines Respirators sicher	2,56	1,35	72,1	27,9
6	Ich fühlte mich sicher, adäquat auf Alarme des Respirators zu reagieren	2,74	1,37	69,1	30,9
7	Ich fühlte mich bei der Vorbereitung einer Infusion sicher	4,74	1,41	21,2	78,8
8	Ich fühlte mich sicher, die Materialien zur Vorbereitung einer ZVK-Anlage selbst zu übernehmen	3,35	1,67	52,9	47,1
9	Ich fühlte mich sicher, die benötigten Materialien für eine Intubation selbst zusammenzustellen	2,63	1,52	77,6	22,4
*Belastung*					
11	Ich fühlte mich durch meine Arbeitsaufgaben oft überfordert	1,81	0,79	95,3	4,7
12	Mein Stresslevel durch die Arbeit war hoch	2,68	1,30	72,1	27,9
13	Ich erlebte Dinge bei meiner Arbeit, die mich psychisch belasteten	2,65	1,34	68,2	31,8
14	Ich hatte Angst, bei meiner Arbeit aufgrund von mangelndem Fachwissen Fehler zu machen	3,06	1,07	62,7	37,3
15	Ich litt unter arbeitsbedingter Erschöpfung	2,16	1,23	85,3	14,7
16	Mein Privatleben litt unter meiner Arbeit	2,34	1,31	80,9	19,1
*Integration ins Team*					
17	Bei meiner Arbeit kannte ich meine Rolle und meine Zuständigkeiten genau	3,72	1,57	42,6	57,4
18	Ich fühlte mich ins Team eingebunden und akzeptiert	4,75	1,38	19,1	80,9
19	Ich fühlte mich von den Patienten akzeptiert	5,38	0,65	0,0	100,0
20	Ich erfuhr von den anderen Teammitgliedern Wertschätzung für meine Arbeit	4,98	1,23	10,6	89,4
21	Bei Fragen oder Unsicherheiten hatten meine Vorgesetzten immer ein offenes Ohr für mich	4,90	1,16	8,8	91,2
22	Fehler und Probleme wurden auf unserer Station nicht vernünftig aufgearbeitet	2,43	1,31	83,6	16,4
*Sinnhaftigkeit*					
23	Ich hatte das Gefühl, durch meine Arbeit etwas Wichtiges und Sinnvolles zu tun	4,66	1,38	17,9	82,1
24	Ich bereue, dass ich mich freiwillig gemeldet habe	1,32	0,89	94,1	5,9
25	Ich empfand meine Arbeit als erfüllend	4,11	1,51	27,3	72,7
*Schulungskonzept*					
26	Die Schulung im AIXTRA hat mich gut auf meinen Arbeitsalltag vorbereitet	3,44	1,27	47,0	53,0

*n* = 168; *t2* nach dem Einsatz auf den Intensivstationen

*Ablehnung* Skalenwerte 1–3; *Zustimmung* Skalenwerte 4–6

*M* Mittelwert, *SD* Standardabweichung

Für die Dimension *Sicherheit* zeigten sich sehr hohe Zustimmungswerte von bis zu 92,6 % für das Sicherheitsgefühl, ein Monitoring bei einem Intensivpatienten anzulegen. Auch bei der Interpretation der Monitoring-Kurven fühlten sich die Studierenden mehrheitlich (88,1 % Zustimmung) sicher, bei der Bedienung und Reaktion auf Alarme eines Respirators jedoch mehrheitlich unsicher (72,1 % und 69,1 % Ablehnung).

In der Dimension *Belastung *waren Ablehnungswerte von 62,7 % bis 95,3 % zu verzeichnen. Die Studierenden waren mehrheitlich nicht überfordert und verspürten keine Angst, Fehler aufgrund von mangelndem Fachwissen zu begehen. Insgesamt war das Belastungsempfinden während des Einsatzes auf der Intensivstation gering.

In der Dimension *Integration ins Team* wurde die wahrgenommene Einbindung sowie Akzeptanz im Team (80,9 % Zustimmung) und ein wertschätzender Umgang (89,4 % Zustimmung) von der überwiegenden Mehrheit positiv bewertet.

94,1 % der Studierenden bereuten es nicht, sich freiwillig für den Einsatz während der COVID-19-Pandemie gemeldet zu haben (Dimension *Sinnhaftigkeit)*.

Das Schulungskonzept als Vorbereitung auf den Arbeitseinsatz wurde uneinheitlich bewertet. Während mehr als die Hälfte der Befragten (53,0 %) angaben, sich durch die Schulung gut auf ihren Arbeitseinsatz vorbereitet gefühlt zu haben, war dies für die übrigen Teilnehmenden (47,0 %) nicht der Fall.

#### Schulungsbewertung und subjektive Sicherheit nach dem Einsatz

Die Analyse zeigt insgesamt 4 signifikante Gruppenunterschiede (Tab. 3). Studierende, die die Schulung als unzureichende Vorbereitung auf ihren Arbeitsalltag wahrnahmen, fühlten sich bei der Vorbereitung einer Infusion (t = 2,31, *p* < 0,05) unsicherer im Vergleich zu Studierenden, die die Schulung gut bewerteten. Gleicher Effekt zeigte sich für das Sicherheitsgefühl an einem intensivmedizinischen Arbeitsplatz (t = 2,20, *p* < 0,05) sowie für die wahrgenommene Sicherheit bei der adäquaten Reaktion auf Monitoring-Alarme (t = 2,66, *p* < 0,05). Am stärksten war der Effekt für das Sicherheitsgefühl bei der Interpretation von Monitoring-Kurven (t = 3,04, *p* < 0,01).

**Table 3:** 

		t2		
Item	Subjektive Selbsteinschätzungen	t	df	*p*
1	Ich fühlte mich sicher, Monitoring (EKG, Sättigung und Blutdruck) bei einem Intensivpatienten anzulegen	1,42	63	0,162
2	Ich fühlte mich sicher, die angezeigten Monitoring-Kurven zu interpretieren	3,04**	63	0,003**
3	Ich fühlte mich sicher, auf Monitoring-Alarme adäquat zu reagieren	2,66*	64	0,010*
4	Ich fühlte mich bei der Arbeit an einem intensivmedizinischen Arbeitsplatz sicher	2,20	64	0,031*
5	Ich fühlte mich bei der Bedienung eines Respirators sicher	1,38	63	0,172
6	Ich fühlte mich sicher, adäquat auf Alarme des Respirators zu reagieren	1,33	63	0,189
7	Ich fühle mich bei der Vorbereitung einer Infusion sicher	2,31	62	0,024*
8	Ich fühle mich sicher, die Materialien zur Vorbereitung einer ZVK-Anlage selbst zu übernehmen	1,21	63	0,233
9	Ich fühle mich sicher, die benötigten Materialien für eine Intubation selbst zusammenzustellen	1,81	62	0,075

*n* = 168; *p<0,05, **p<0,01; *t2* Nach dem Einsatz auf den Intensivstationen

Die Schulung im AIXTRA hat mich gut auf meinen Arbeitsalltag vorbereitet:* Ablehnung* Skalenwerte 1–3; *Zustimmung* Skalenwerte 4–6

*t* Prüfgrößenwert des t‑Tests für unabhängige Stichproben, *df* Freiheitsgrade

## Diskussion

Das innovative und umfassende Konzept [7] ist eine effiziente und wirksame Methode zu Rekrutierung, Schulung sowie Einsatzplanung von Medizinstudierenden, um medizinisches Personal in Ausnahmesituationen zu entlasten.

### Rekrutierung.

Kurzfristig konnten über 250 Medizinstudierende mit einer Mindeststudiendauer von 8 Semestern für den Einsatz auf der Intensivstation rekrutiert und geschult werden, um das medizinische System während der Coronapandemie zu unterstützen. Die Erfahrungen der vorliegenden Arbeit zeigen, dass die Rekrutierung einer Vielzahl an freiwilligen Studierenden zur Unterstützung des medizinischen Systems während Pandemien problemlos möglich ist.

### Schulung.

Die Daten belegen eindeutig die kurzfristige Machbarkeit und Umsetzbarkeit der Schulungsmaßnahme, die einen positiven Effekt auf das Sicherheitsgefühl der Medizinstudierenden hatte. Diese fühlten sich im Hinblick auf ihren Einsatz auf den Intensivstationen, insbesondere bei der Ausübung trainierter medizinischer Fertigkeiten, sicherer. Dies verdeutlicht, dass das Schulungsmodul ein wichtiger Bestandteil des Gesamtkonzeptes und für die Einsatzvorbereitung unerlässlich ist. Insbesondere dient es dazu, Berührungsängste und Unsicherheiten abzubauen. Eine Auswirkung eines evtl. fälschlichen Sicherheitsgefühls auf die Patientenversorgung kann hierbei nicht ausgeschlossen werden, erscheint jedoch aus verschiedenen Gründen unwahrscheinlich. Zum einen übten die Studierenden lediglich assistierende Tätigkeiten unter Aufsicht von examinierten Pflegekräften aus, sodass die Wahrscheinlichkeit sicherheitsrelevanter unbemerkter Fehler sehr gering war. Zum anderen ist davon auszugehen, dass evtl. fehlerhafte Annahmen hinsichtlich der Abläufe in der Praxis schnell korrigiert wurden (z. B. durch Feedback seitens der Praxisanleitenden).

Die Festlegung der Kursdauer erfolgte nach Abwägung verschiedener Aspekte. Zum einen erschienen ein Theorieteil von 2,5 h sowie ein ebenso langer Praxisteil mit einer Wechselzeit von ca. 30 min/Station als inhaltlich ausreichend, um die freiwilligen Helfenden adäquat auf ihren Einsatz vorzubereiten. Zum anderen handelte es sich um eine freiwillige Veranstaltung, sodass der gewählte Zeitumfang nicht nur inhaltlich angemessen, sondern auch zumutbar sein sollte. Neben der Abwägung von zu vermittelnden Inhalten sowie der Freiwilligkeit, die Veranstaltung zu besuchen, spielte jedoch auch das Infektionsrisiko eine Rolle in der Wahl der Kursdauer und des Kursumfangs. Um das Infektionsrisiko möglichst gering zu halten, wurde von einer Aufteilung der Veranstaltung auf verschiedene Tage abgesehen. Die 5‑stündige Schulung hatte keinesfalls den Anspruch, eine adäquate und suffiziente Einarbeitung in die Bedienung von Respiratoren bei kritisch kranken Patienten zu vermitteln.

### Einsatz.

Es ist schwer abschätzbar, wie viel zusätzliches medizinisches Personal zum Zeitpunkt der ersten Pandemiewelle in Deutschland benötigt wurde. Ausgehend von 36 medizinischen Fakultäten und der Möglichkeit, mit selbigem Konzept an diesen universitären Standorten Medizinstudierende zu rekrutieren und zu schulen, ergäbe sich eine Anzahl von 9000 zusätzlichen medizinischen Helfenden für das gesamte medizinische System in Deutschland.

Nicht alle geschulten Studierenden konnten dem System zugeführt werden. Die Gründe hierfür sind vielfältig und reichen von rechtlichen Aspekten über Entgeltregelungen bei fehlenden Tarifverträgen sowie Schweigepflicht und Verschwiegenheitsklauseln bis hin zu administrativen Ursachen. Zur Vorbereitung zukünftiger Einsätze empfehlen wir, diese Aspekte für jeden Standort individuell zu prüfen, rechtliche Hürden frühzeitig zu identifizieren sowie Verantwortlichkeiten im administrativen Prozess zu klären. Bei Letzterem kann eine Standard Operating Procedure (SOP) wertvolle Zeit einsparen.

Geschultes, aber nichteingesetztes Assistenzpersonal kann anderen Krankenhäusern als Ressource zur personellen Unterstützung dienen. Daher kommt dieses Konzept nicht nur universitären Standorten zugute, sondern allen medizinischen Einrichtungen.

Hinsichtlich des Einsatzes sind insbesondere das niedrige Belastungsempfinden und die hohe Akzeptanz sowie Wertschätzung innerhalb der Teams als sehr positiv zu betrachten. Insgesamt ist der Einsatz im Rahmen dieses Konzeptes auch für die Studierenden zumutbar und verantwortbar.

### Weiterentwicklungsbedarf

Aus den Ergebnissen wird ein Weiterentwicklungsbedarf am Schulungsmodul deutlich, da sich das Sicherheitsgefühl der Studierenden je nach medizinischer Tätigkeit deutlich unterscheidet. Zum einen fühlten sich die meisten Studierenden bei der Bedienung und Reaktion auf Alarme von Respiratoren unsicher, dennoch hatte dies keinen signifikanten Einfluss auf die Bewertung der Schulung. Ein Grund dafür könnte sein, dass die Studierenden primär assistierende Aufgaben übernehmen sollten und die Bedienung der Beatmungsgeräte medizinischem Fachpersonal vorbehalten war. Die Studierenden konnten das erlernte theoretische Wissen möglicherweise nicht bei jeder Tätigkeit in gleichem Maße in der Praxis anwenden. Außerdem ist der Umgang mit diesen selbst für Fachkräfte, die deutlich länger und intensiver auf die Bedienung von Respiratoren vorbereitet werden, ein Angstthema [13]. Dennoch war das Erlernen der Bedienung eines Respirators aus Gründen der psychologischen Sicherheit wichtig und erwünscht. Dies wurde bei der Schulungsplanung berücksichtigt und im Expertenteam diskutiert und entschieden. Insbesondere war das Ziel, mögliche Hemmungen vor Technik abzubauen, die Studierenden auf ein Umfeld mit erhöhtem Stressniveau vorzubereiten und um das Sicherheitsgefühl am eigentlichen Einsatzbereich zu stärken. Es ist zu prüfen, ob die Bedienung eines Respirators aus Kurzschulungen für den ICU-Einsatz entfernt werden sollte.

Zum anderen liegt der identifizierte Weiterentwicklungsbedarf in den Bereichen Monitoring und Infusionsvorbereitung, in denen sich bis zu einem Viertel der Befragten (23,5 %) nach dem Einsatz unsicher fühlte. Dies korrespondierte mit der Bewertung des Schulungskonzeptes. Die Infusionsvorbereitung und Beurteilung von Monitoring-Kurven sind von großer klinischer Relevanz und könnten bei fehlerhafter Interpretation und Durchführung weitreichende Folgen für Patient*innen haben. Folglich gilt es, dies intensiver zu schulen und genauer zu erfragen, welche spezifischen Tätigkeiten die Studierenden hinsichtlich dieser Themen in der Praxis bearbeiteten. So lassen sich der klinische Bedarf und somit auch der Schulungsbedarf genauer ermitteln. Eine Möglichkeit zur Weiterentwicklung der Schulungsmaßnahme und folglich zur Entlastung der Pflegekräfte könnte beispielsweise die MPG-konforme Einweisung auf Spritzen- bzw. Infusionspumpen sein. Dasselbe gilt für die Praxismodule „ICU Arbeitsplatz“ und „ICU Monitoring Vitaldaten“.

Bestehender Nachschulungsbedarf sollte frühzeitigt erkannt werden. Studierende, die sich hinsichtlich bestimmter Fertigkeiten unsicher fühlen, sollten die Möglichkeit zu Nachschulungen erhalten. Darüber hinaus sollten sich zukünftige Studien mit Langzeiteffekten von Schulungsmaßnahmen beschäftigen und Aufschluss über den langfristigen Weiterbildungsbedarf von medizinischem Personal während Pandemiebedingungen liefern.

Es wird ausdrücklich betont, dass dieses Konzept keinesfalls dazu dient, (Fach)-Pflegende durch Medizinstudierende zu ersetzen. Es ist darauf ausgelegt, diese in besonderen Situationen durch assistierende Aufgaben zu unterstützen, und eignet sich auch für Szenarien abseits von Pandemien, bei denen größere Personalkapazitäten innerhalb kürzester Zeit akquiriert und für den zeitnahen Einsatz im medizinischen System qualifiziert werden müssen (z. B. Naturkatastrophen, atomare Katastrophen usw.).

### Limitationen

Aus ethischen Aspekten konnte die Etablierung einer Kontrollgruppe nicht ermöglicht werden. Es ist nicht zu verantworten, Studierende in einer Pandemiesituation mit einer hochansteckenden und potenziell schwer verlaufenden Erkrankung ohne entsprechende Schulung auf Intensivstationen einzusetzen.

Im Anschluss an die Datenerhebung fiel (dank eines Reviewer-Hinweises) eine unglückliche Formulierung bei Item 8 der Sicherheitsdimension auf. Treffender wäre die Formulierung „Ich fühle mich sicher, die Vorbereitung der Materialien für eine ZVK-Anlage selbst zu übernehmen“ gewesen. Es ist davon auszugehen, dass die Befragten dieses Item auch dementsprechend verstanden haben; allerdings kann dies nicht mit Gewissheit nachvollzogen werden.

Des Weiteren ist die konkrete Verwendung der Helfenden auf Station unbekannt, da eine genaue Rückverfolgung, welche spezifischen Tätigkeiten die Studierenden in ihrem jeweiligen Einsatz durchführten, nicht Bestandteil dieser Studie war. Zukünftige Studien sollten dies berücksichtigen, um Aussagen über die konkrete Verwendung der Helfenden treffen zu können. Darüber hinaus befasst sich die vorliegende Studie lediglich mit den Bewertungen des eingesetzten Hilfspersonals, nicht jedoch mit den Einschätzungen der regulären Pflegekräfte. Zukünftige Studien sollten auch eine Einsatzbewertung durch das reguläre Pflegepersonal einbeziehen, um die Ergebnisse und den Mehrwert durch freiwillige Helfende in einen Gesamtzusammenhang setzen zu können.

## Resümee

Die Wahrscheinlichkeit weiterer Wellen und zukünftiger Pandemien ist sehr hoch [14–16]. Daher bedarf es ganzheitlicher Konzepte, um potenziell auftretenden Personalengpässen in der medizinischen Versorgung zu begegnen. Das evaluierte, innovative Konzept zu zielgerichteter und effizienter Rekrutierung, Schulung sowie Einsatzplanung von Medizinstudierenden leistet einen wichtigen Beitrag, um das deutschlandweite medizinische System zu unterstützen.

## Fazit für die Praxis

- Das innovative und umfassende Konzept ist eine effiziente und wirksame Methode zu Rekrutierung, Schulung sowie Einsatzplanung von Medizinstudierenden als zusätzliches medizinisches Personal in Ausnahmesituationen.
- Durch das integrierte Schulungsmodul fühlen sich die Medizinstudierenden deutlich besser auf einen Einsatz auf der Intensivstation während der COVID-19-Pandemie vorbereitet.
- Hinsichtlich spezifischer Schulungsmaßnahmen für den Einsatz von Medizinstudierenden in Ausnahmesituationen besteht sowohl Forschungs- als auch Weiterentwicklungsbedarf.

## Supplementary Information







