# Supplementary material for: Medical students as helpers in the pandemic: Innovative concept for recruitment, training and assignment planning of medical students as medical personnel during the COVID-19 pandemic
Source: Anaesthesist. 2021 Jul 20;71(1):21–9. [Article in German] doi: 10.1007/s00101-021-01009-3 (PMC8290386; doi:10.1007/s00101-021-01009-3)
Supplement: Supplementary file 1 [file 101_2021_1009_MOESM1_ESM.pdf]

Corona-Schulung Studierende Prä

Datum:

Probandencode:

Umfrage erstellt mit
LamaPoll

	trifft überhaupt nicht zu					trifft voll zu
Ich erhoffe mir viel von der Fortbildung .	○	○	○	○	○	○
Ich sehe die Fortbildung als Chance, etwas Neues zu lernen.	○	○	○	○	○	○
Eigentlich habe ich keine Lust auf die Fortbildung.	○	○	○	○	○	○
Praktische Schulungsmaßnahmen durch klinische Simulation bringen mir nichts, weil im wirklichen Leben sowieso alles anders ist.	○	○	○	○	○	○
Zeit und Geld für praktische Schulungsmaßnahmen im Schulungs-/Simulationszentrum sollte man besser in sinnvollere Dinge investieren.	○	○	○	○	○	○
Praktische Trainings/Simulationstrainings kommen letztlich den Patientinnen und Patienten zugute.	○	○	○	○	○	○
Ich halte Simulation für ein geeignetes Mittel zum Training praktischer Fertigkeiten.	○	○	○	○	○	○
Andere Formen des Lernens halte ich für viel sinnvoller als Simulation.	○	○	○	○	○	○
Ich fühle mich bereit, Monitoring (EKG, Sättigung und Blutdruck) beim Intensivpatienten anzulegen.	○	○	○	○	○	○
Ich fühle mich bereit, die angezeigten Monitoring-Kurven zu interpretieren.	○	○	○	○	○	○
Ich fühle mich bereit, auf Monitoring-Alarme adäquat zu reagieren.	○	○	○	○	○	○

Umfrage erstellt mit
LamaPoll

Ich fühle mich bei der Arbeit an einem intensivmedizinischen Arbeitsplatz sicher.	○	○	○	○	○	○
Ich fühle mich bei der Bedienung eines Respirators sicher.	○	○	○	○	○	○
Ich fühle mich bereit, adäquat auf Alarme des Respirators zu reagieren.	○	○	○	○	○	○
Ich fühle mich bei der Vorbereitung einer Infusion sicher.	○	○	○	○	○	○
Ich fühle mich bereit, einen Bettplatz auf Normalstation eigenständig vorzubereiten.	○	○	○	○	○	○
Ich fühle mich bereit, die Materialien zur Vorbereitung einer ZVK-Anlage selbst zu übernehmen.	○	○	○	○	○	○
Ich fühle mich bereit, die benötigten Materialien für eine Intubation selbst zusammenzustellen.	○	○	○	○	○	○

Ich habe in der Vergangenheit bereits eigene Erfahrungen mit Simulationstrainings gemacht.

○ Ja ○ Nein

	Gar keine Rolle					Eine wichtige Rolle
Bei meiner jetzigen Arbeit / Ausbildung spielt Simulation...	○	○	○	○	○	○

Umfrage erstellt mit
LamaPoll
