# Supplementary material for: Medical students as helpers in the pandemic: Innovative concept for recruitment, training and assignment planning of medical students as medical personnel during the COVID-19 pandemic
Source: Anaesthesist. 2021 Jul 20;71(1):21–9. [Article in German] doi: 10.1007/s00101-021-01009-3 (PMC8290386; doi:10.1007/s00101-021-01009-3)
Supplement: Supplementary file 2 [file 101_2021_1009_MOESM2_ESM.pdf]

Corona-Schulung Studierende Post

Datum:

Probandencode:

Umfrage erstellt mit
LamaPoll

	trifft überhaupt nicht zu					trifft voll zu
Ich habe mich wohl gefühlt, die praktische Übung/Simulation in der Gruppe zu machen.	○	○	○	○	○	○
Ich habe zwischenzeitlich fast vergessen, dass die Praxistätigkeit/Situation nur simuliert war.	○	○	○	○	○	○
Ich konnte mich gut auf die Praxisübung/Simulation einlassen.	○	○	○	○	○	○
Ich habe durch die gesamte Schulung (Theorie + Praxisübung/Simulation) etwas Wichtiges und Sinnvolles gelernt.	○	○	○	○	○	○
Was ich in der gesamten Schulung (Theorie + Praxisübung/Simulation) gelernt habe, brauche ich auch in meinem Arbeitsalltag.	○	○	○	○	○	○
Praktische Schulungsmaßnahmen durch klinische Simulation bringen mir nichts, weil im wirklichen Leben sowieso alles anders ist.	○	○	○	○	○	○
Zeit und Geld für praktische Schulungsmaßnahmen im Schulungs-/Simulationszentrum sollte man besser in sinnvollere Dinge investieren.	○	○	○	○	○	○
Praktische Trainings/Simulationstrainings kommen letztlich den Patientinnen und Patienten zugute.	○	○	○	○	○	○
Ich halte Simulation für ein geeignetes Mittel zum Training praktischer Fertigkeiten.	○	○	○	○	○	○
Andere Formen des Lernens halte ich für viel sinnvoller als Simulation.	○	○	○	○	○	○

Umfrage erstellt mit LamaPoll

Ich fühle mich bereit, Monitoring (EKG, Sättigung und Blutdruck) beim Intensivpatienten anzulegen. ○ ○ ○ ○ ○ ○

Ich fühle mich bereit, die angezeigten Monitoring-Kurven zu interpretieren. ○ ○ ○ ○ ○ ○

Ich fühle mich bereit, auf Monitoring-Alarme adäquat zu reagieren. ○ ○ ○ ○ ○ ○

Ich fühle mich bei der Arbeit an einem intensivmedizinischen Arbeitsplatz sicher. ○ ○ ○ ○ ○ ○

Ich fühle mich bei der Bedienung eines Respirators sicher. ○ ○ ○ ○ ○ ○

Ich fühle mich bereit, adäquat auf Alarme des Respirators zu reagieren. ○ ○ ○ ○ ○ ○

Ich fühle mich bei der Vorbereitung einer Infusion sicher. ○ ○ ○ ○ ○ ○

Ich fühle mich bereit, einen Bettplatz auf Normalstation eigenständig vorzubereiten. ○ ○ ○ ○ ○ ○

Ich fühle mich bereit, die Materialien zur Vorbereitung einer ZVK-Anlage selbst zu übernehmen. ○ ○ ○ ○ ○ ○

Ich fühle mich bereit, die benötigten Materialien für eine Intubation selbst zusammenzustellen. ○ ○ ○ ○ ○ ○

Insgesamt fand ich die Praxisübung/Simulation...

Sehr schlecht					Sehr gut
○	○	○	○	○	○

Umfrage erstellt mit
LamaPoll

Das Thema der Praxisübung/Simulation hat mich...

Gar nicht interessiert ○ ○ ○ ○ ○ ○ Sehr interessiert

Folgende Aspekte waren besonders relevant und hilfreich:

Folgende Aspekte haben gefehlt oder sind zu kurz gekommen:

Folgende Aspekte haben mir nicht gefallen:

Umfrage erstellt mit
LamaPoll
