# Supplementary material for: Medical students as helpers in the pandemic: Innovative concept for recruitment, training and assignment planning of medical students as medical personnel during the COVID-19 pandemic
Source: Anaesthesist. 2021 Jul 20;71(1):21–9. [Article in German] doi: 10.1007/s00101-021-01009-3 (PMC8290386; doi:10.1007/s00101-021-01009-3)
Supplement: Supplementary file 3 [file 101_2021_1009_MOESM3_ESM.pdf]

Kompetenzzentrum AIXTRA Training und Patientensicherheit

Umfrage zum Einsatz von Studierenden während der Corona-Pandemie

Liebe Studierende,

vielen Dank, dass Sie sich die Zeit nehmen, diesen Fragebogen auszufüllen.
Dieser Fragebogen fragt nach Ihren Meinungen rund um Ihren Einsatz als Pflegehelfer/in auf den Intensivstationen im Rahmen der Covid-19-Pandemie.
Die Bearbeitung dauert etwa 10 Minuten.
Die Teilnahme an der Umfrage ist **freiwillig**. Wir hoffen jedoch auf viele Teilnehmer*innen, um aussagekräftige Ergebnisse generieren zu können.
Die Erhebung erfolgt **anonym**. Niemand wird Ihre Antworten Ihrer Person zuordnen können. Aus Zuordnungsgründen erstellen Sie einen Probandencode, der nur Ihnen bekannt ist.
Der Fragebogen möchte nur Ihre Vorstellungen und Meinungen erfragen. Es gibt keine richtigen oder falschen Antworten.
Für weitere Fragen stehen wir Ihnen gerne zur Verfügung.
Vielen herzlichen Dank für die Teilnahme!

Das AIXTRA-Team

Ansprechpartner:
Dr. Saša Sopka, MME, Leiter AIXTRA – Kompetenzzentrum für Training und Patientensicherheit, Tel.: 0241/80 35355, ssopka@ukaachen.de
Kim Pears, AIXTRA – Kompetenzzentrum für Training und Patientensicherheit, kpears@ukaachen.de
Michelle Schmidt, AIXTRA – Kompetenzzentrum für Training und Patientensicherheit, mischmidt@ukaachen.de

Umfrage erstellt mit
LamaPoll

★ Probandencode

Bitte tragen Sie hier aus Zuordnungsgründen Ihren **individuellen Code** nach folgendem Schema ein:

Anfangsbuchstabe Vorname Ihrer Mutter
Anfangsbuchstabe Vorname Ihres Vaters
Geburtstag Ihrer Mutter
Geburtstag Ihres Vaters

Beispiel:
Vorname Mutter: Anna => A
Vorname Vater: Max => M
Geburtstag Mutter: 05. März => 05
Geburtstag Vater: 12. November => 12
Beispielcode: AM0512

Individueller Code

Umfrage erstellt mit
LamaPoll

Fachsemester

Ich studiere im Sommersemester 2020 im Fachsemester...

- ◯ 1
- ◯ 2
- ◯ 3
- ◯ 4
- ◯ 5
- ◯ 6
- ◯ 7
- ◯ 8
- ◯ 9
- ◯ 10
- ◯ 11
- ◯ 12
- ◯ >12

Ausbildung

Ich habe vor dem Medizinstudium bereits eine Ausbildung im medizinischen Bereich abgeschlossen.

◯ Ja ◯ Nein

Umfrage erstellt mit
LamaPoll

Falls ja, welche Ausbildung?

Welche Ausbildung haben Sie abgeschlossen?

Umfrage erstellt mit
LamaPoll

Bitte bewerten Sie folgende Aussagen:

Bitte wählen Sie für jede Aussage die für Sie zutreffende Antwortmöglichkeit aus.

	Stimme überhaupt nicht zu					Stimme voll zu
Ich fühlte mich durch meine Arbeitsaufgaben oft überfordert.	○	○	○	○	○	○
Mein Stresslevel durch die Arbeit war hoch.	○	○	○	○	○	○
Ich erlebte Dinge bei meiner Arbeit, die mich psychisch belasteten.	○	○	○	○	○	○
Ich hatte Angst, bei meiner Arbeit aufgrund von mangelndem Fachwissen Fehler zu machen.	○	○	○	○	○	○
Bei meiner Arbeit kannte ich meine Rolle und meine Zuständigkeiten genau.	○	○	○	○	○	○
Ich fühlte mich ins Team eingebunden und akzeptiert.	○	○	○	○	○	○
Ich fühlte mich von den Patienten akzeptiert.	○	○	○	○	○	○
Ich erfuhr von den anderen Teammitgliedern Wertschätzung für meine Arbeit.	○	○	○	○	○	○
Ich litt unter arbeitsbedingter Erschöpfung.	○	○	○	○	○	○
Ich empfand meine Arbeit als erfüllend.	○	○	○	○	○	○
Mein Privatleben litt unter meiner Arbeit.	○	○	○	○	○	○
Die Schulung im AIXTRA hat mich gut auf meinen Arbeitsalltag vorbereitet.	○	○	○	○	○	○
Bei Fragen oder Unsicherheiten hatten meine Vorgesetzten immer ein offenes	○	○	○	○	○	○

Umfrage erstellt mit
LamaPoll

Ohr für mich.

Fehler und Probleme wurden auf unserer Station nicht vernünftig aufgearbeitet.	◯	◯	◯	◯	◯	◯
Ich hatte das Gefühl, durch meine Arbeit etwas Wichtiges und Sinnvolles zu tun.	◯	◯	◯	◯	◯	◯
Ich bereue, dass ich mich freiwillig gemeldet habe.	◯	◯	◯	◯	◯	◯
Ich fühlte mich sicher, Monitoring (EKG, Sättigung und Blutdruck) beim Intensivpatienten anzulegen.	◯	◯	◯	◯	◯	◯
Ich fühlte mich sicher, die angezeigten Monitoring-Kurven zu interpretieren.	◯	◯	◯	◯	◯	◯
Ich fühtle mich sicher, auf Monitoring-Alarme adäquat zu reagieren.	◯	◯	◯	◯	◯	◯
Ich fühlte mich bei der Arbeit an einem intensivmedizinischen Arbeitsplatz sicher.	◯	◯	◯	◯	◯	◯
Ich fühlte mich bei der Bedienung eines Respirators sicher.	◯	◯	◯	◯	◯	◯
Ich fühlte mich sicher, adäquat auf Alarme des Respirators zu reagieren.	◯	◯	◯	◯	◯	◯
Ich fühlte mich bei der Vorbereitung einer Infusion sicher.	◯	◯	◯	◯	◯	◯
Ich fühlte mich sicher, einen Bettplatz auf Normalstation eigenständig vorzubereiten.	◯	◯	◯	◯	◯	◯
Ich fühlte mich sicher, die Materialien zur Vorbereitung einer ZVK-Anlage selbst zu übernehmen.	◯	◯	◯	◯	◯	◯
Ich fühlte mich sicher, die benötigten Materialien für eine Intubation selbst zusammenzustellen.	◯	◯	◯	◯	◯	◯

Umfrage erstellt mit LamaPoll

Anregungen & Wünsche

Sollte ein weiterer Einsatz notwendig werden, würde ich mir folgende Themen für eine Schulung wünschen:

Für meine Arbeit auf der Station hätte ich mir gewünscht, dass ...

Umfrage erstellt mit
LamaPoll
