# Supplementary figures and images for: Medical students as helpers in the pandemic: Innovative concept for recruitment, training and assignment planning of medical students as medical personnel during the COVID-19 pandemic
Source: Anaesthesist. 2021 Jul 20;71(1):21–9. [Article in German] doi: 10.1007/s00101-021-01009-3 (PMC8290386; doi:10.1007/s00101-021-01009-3)

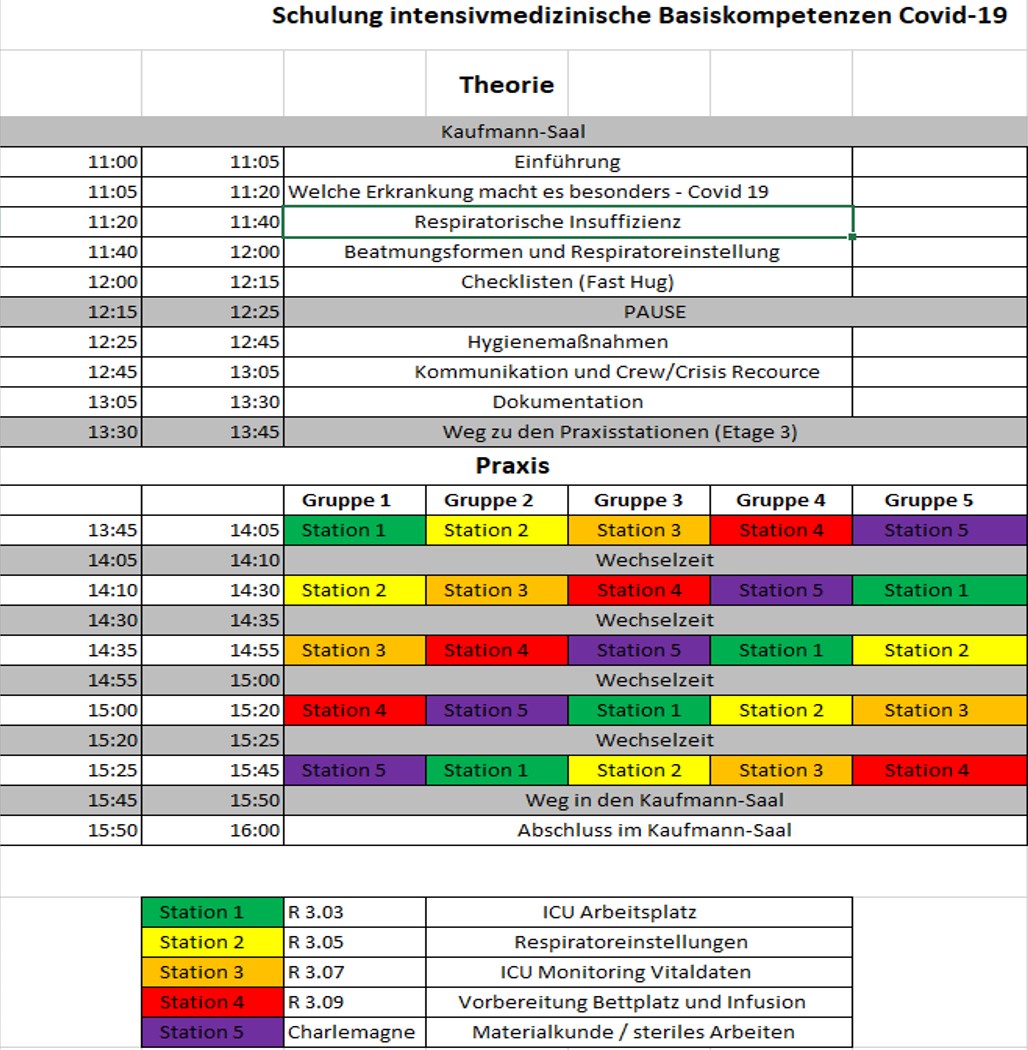

Supplement: Supplementary file 4 [file 101_2021_1009_MOESM4_ESM.jpg]
